# *Aeromonas salmonicida*: Genomics, Taxonomy, Diversity, Pathogenesis, Treatments and Beyond

**DOI:** 10.3390/microorganisms11051189

**Published:** 2023-05-01

**Authors:** Steve J. Charette

**Affiliations:** 1 Département de Biochimie, de Microbiologie et de Bio-Informatique, Faculté des Sciences et de Génie, Université Laval, Quebec City, QC G1V 0A6, Canada; steve.charette@bcm.ulaval.ca; 2 Institut de Biologie Intégrative et des Systèmes, Pavillon Charles-Eugène-Marchand, Université Laval, Quebec City, QC G1V 0A6, Canada; 3 Centre de Recherche de l’Institut Universitaire de Cardiologie et de Pneumologie de Québec, Quebec City, QC G1V 4G5, Canada

For a long time, the bacterial species *Aeromonas salmonicida* seemed to be limited to a regrouping of psychrophilic subspecies that infect fish, particularly salmonids. However, the description of the mesophilic subspecies *pectinolytica* in the early 2000s represented a major upheaval in our knowledge of this bacterial species [1]. Since then, thanks to the use of omics methods, the species *A. salmonicida* has revealed part of its greatly underestimated diversity. A perspective article on this subject opens this Special Issue, entitled *Aeromonas salmonicida*: Genomics, Taxonomy, Diversity, Pathogenesis, Treatments and Beyond [2]. The introductory article describes the new dichotomy found within the species *A. salmonicida*; on one side are the four classically described psychrophilic subspecies (*salmonicida*, *smithia*, *achromogenes*, and *masoucida*), and on the other are an increasing number of mesophilic strains that are capable of growing at temperatures of 37 °C and even higher, but also at lower temperatures like psychrophilic strains. These mesophilic strains, some of which have been described recently [3,4], present a much greater taxonomic diversity than psychrophilic strains, which goes hand in hand with the multitude of environments and hosts from which these mesophilic strains originate. The perspective article prompts us to reconsider our view of the taxonomy of the species *A. salmonicida* and to remain attentive to the presence of mesophilic strains of this species in unexpected environments. 

Learning more about mesophilic strains of *A. salmonicida* is necessary to obtain a complete portrait of this bacterial species. Chen et al. contributed to a better understanding of the characteristics of the mesophilic strain SRW-OG1, isolated from a warm-water fish, that may have pathogenic potential in humans [5]. This is described in an article included in this Special Issue [6]. In their study, the authors looked at the transcriptome of the SRW-OG1 strain grown at 18, 28, and 37 °C. The *aerA* and *hlyA* genes for aerolysin and hemolysin, two important virulence factors, were found to be significantly upregulated at 28 and 37 °C. Higher temperatures are also associated with increased hemolytic activity. This study demonstrated that the virulence of the SRW-OG1 strain is closely linked to the culture temperature.

The taxonomy of *A. salmonicida* is discussed in another article in the Special Issue by the team of J. Santander [7]. In this case, the focus is on the analysis of the complete genome of atypical psychrophilic strains. Notably, the abundance of repeated sequences in the genome, especially in psychrophilic strains, makes it impossible to obtain a complete closed genome using short-read, high-throughput sequencing technologies [8]. Thus, even though the number of genomes in the databases is increasing, the majority of these are in the form of contigs only. To help to better understand the diversity and pathogenesis, Vasquez et al. produced the complete genome of four psychrophilic strains of *A. salmonicida*, including three with atypical characteristics (J409, J410, J411) [7]. Obtaining these genomes enabled the authors to make a very detailed analysis of their characteristics, including their phylogeny, chromosome synteny, presence of insertion sequences, virulence and transcriptomic regulation genes, non-coding RNA, and plasmids. The authors propose that the classification of psychrophilic subspecies reflects their ecological niche and accessory genome more than divergences in their core genome.

In a second article published in this Special Issue, J. Santander’s team explored the ability of the typical psychrophilic strain J223, whose genome was studied in the previous article [7], to cause infection in lumpfish [9]. This study focused more specifically on the expression of lumpfish genes in the blood, head kidney, spleen, and liver during infection. The authors proposed a model where *A. salmonicida* could induce fish death through blood clotting, complement activation, and inflammation, leading to various consequences such as hypoxia and hemorrhaging of internal organs. In addition, *A. salmonicida* acts negatively on the structure of the host cell cytoskeleton by depolymerizing actin and microtubules to colonize and survive inside the lumpfish while disrupting signaling pathways such as NF-kB.

Last but not least, the final article included in this Special Issue addresses the question of the proteome of *A. salmonicida* subsp. *salmonicida* when facing various stresses: florfenicol (an antibiotic), a higher culture temperature (19 °C compared to 13 °C), or a limitation of iron in the culture medium [10]. Several iron-regulated proteins that had not previously been reported in the literature for *A. salmonicida* were identified in this study of the psychrophilic strain JF2267. In addition, several proteins were frequently more abundant both in iron-limiting conditions and at higher temperatures. The proteome of outer membrane vesicles (OMVs) is also disturbed by the stresses tested, suggesting a role of these vesicles in the acquisition of iron. The authors even propose that the OMVs produced in iron-limiting conditions could be used as potential vaccines to allow fish to develop an immune response.

These five articles demonstrate that omics methods provide immense advantages for the study of the evolution, diversity, and virulence of *A. salmonicida*. Three of these articles also reveal the close link that exists between temperature and this bacterial species, whether through the dichotomy between psychrophilic and mesophilic strains or the impact of a variation in temperature on the expression of the pathogenic phenotype of each group. Several new avenues have recently been opened for the study of *A. salmonicida*, and the articles in this Special Issue constitute a very interesting selection for those who want a quick overview of the state of research on this bacterial species.
